# What do laboratories know about the pneumatic tube delivery systems they depend on?

**DOI:** 10.11613/BM.2026.030801

**Published:** 2026-08-10

**Authors:** Anushka Jayanetti, Tony Badrick

**Affiliations:** Royal College of Pathologists of Australasia Quality Assurance Programs, St Leonards, New South Wales, Australia

**Keywords:** quality assurance, preanalytic error, specimen quality

## Abstract

**Introduction:**

Pneumatic tube systems (PTS) are a key component of many laboratory sample delivery systems. However, they can be a source of preanalytical error, particularly when samples are lost or damaged. The Key Incident Monitoring and Management (KIMMS) program of the Royal College of Pathologists Australasia Quality Assurance Programs (RCPAQAP) conducted a survey to gather information on the maintenance of these systems.

**Materials and methods:**

The categories of questions included general information, service, maintenance and validation of systems, and incident reporting. The responses were compiled and reviewed using Microsoft Forms.

**Results:**

The survey found a lack of awareness of servicing and validation records, as well as of the PTS’s functionality. Most sites reported incidents with their pneumatic tube systems (37/45, 80%), mainly system failures rather than samples being lost, delayed, or damaged.

**Conclusions:**

Greater oversight of laboratories is needed, and relationships with system vendors must be strengthened to ensure quality service.

## Graphical abstract

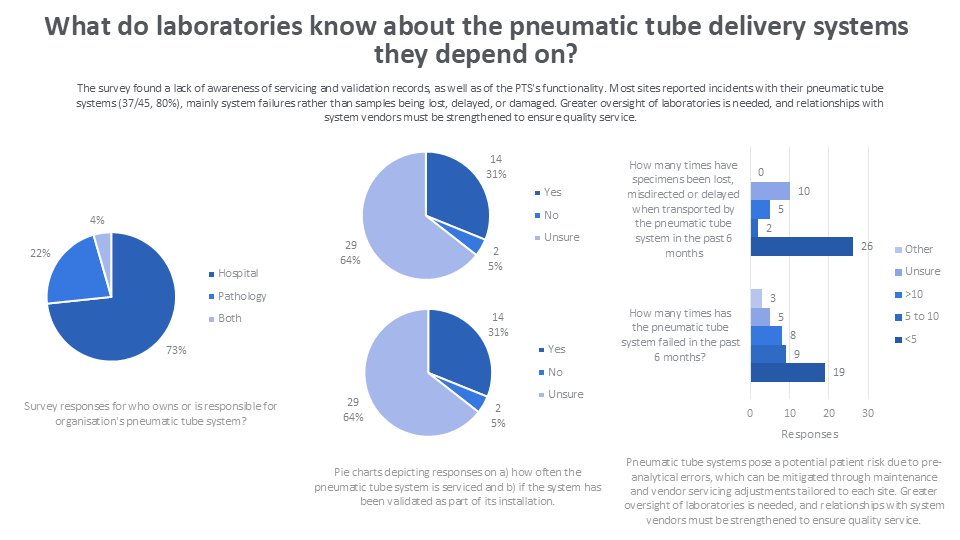

## Highlights

• Survey of laboratories on pneumatic tube system governance

• Half of the respondents were unsure how often their pneumatic tube systems were serviced

• Total of 63% didn’t know if the systems were validated before installation

## Introduction

Pneumatic tube systems (PTS) are extensively used within clinical laboratories to transport patient samples, paperwork, and pharmacy items within or between organisations, and, to a lesser extent, blood products (*1*). Compared to manual transport, PTS have been shown to improve turnaround times and do not increase hemolysis (*2*-*4*). Some measurands may be impacted by the rapid acceleration in the PTS, such as platelet-related tests (*5*). Lactate dehydrogenase (LD), potassium, D-dimer, and prothrombin time have been shown to be affected by PTS (*1*, *6*, *7*). Also, blood gas samples should not be transported in PTS, and contradicting data exists on the transport of cerebrospinal fluid specimens for the diagnosis of hemorrhage. Adequate centrifugation of samples is essential, or any cells left above the gel plug can hemolyse, dependent on time and speed of the PTS (*4*). However, all these papers assume that the PTS is operating optimally and regularly serviced.

Methods for validating PTS have been proposed using the relationship between analyte concentrations in plasma, such as LD and potassium, and the PTS forces recorded by 3-axis accelerometers, or by more formal procedures that are not widely used in Australia (*6*, *8*). There has been little in the literature on the governance of PTS, which is important when the system may be shared between a laboratory and other hospital units.

Feedback from the 2023 Royal College of Pathologists Australasia Quality Assurance Programs (RCPAQAP) The Key Incident Monitoring and Management (KIMMS) workshop indicated that the maintenance and performance of pneumatic tube systems are not always optimal and may lead to preanalytical errors, such as hemolysed or leaked samples. In the KIMMS program, incidents related to PTS can be reported under the general transport delay category, but there is no specific PTS category. This research sought to identify issues relating to the governance and maintenance of PTS used by clinical laboratories. Therefore, for this project, we aimed to collect information on pneumatic tube systems utilised by, and often shared by, clinical laboratories to assess how they were validated and managed.

## Materials and methods

We circulated a Microsoft Forms (Microsoft Corporation, Redmond, USA) audit *via* email to 89 quality managers across Australia/New Zealand. The survey is included in the Supplementary data. The audit was developed under the guidance of the RCPAQAP KIMMS advisory committee. Responses were anonymous, but an email address was requested. No ethical approval or institutional oversight was required. The categories of questions included general information, service, maintenance and validation of systems, and incident reporting. The responses were compiled and reviewed using Microsoft Forms. There was no statistical analysis – the data and graphs were produced by the Microsoft Forms software.

## Results

Forty-five responses (51% response rate) were received from public and private pathology, with some respondents submitting multiple forms if their site had several PTS. Most of the laboratories (78%, 35/45) were part of networks of clinical laboratories, while 16% (7/45) operated as stand-alone laboratories. The remaining 6% (3/45) were laboratories that were part of a larger non-laboratory organisation. Regarding the age of these systems, 30% (14/45) had been in use for 11-20 years, 22% (10/45) for 6-10 years, 15% (7/45) for over 21 years, and 11% (5/45) for under 5 years. Only 4% (1/45) of respondents had systems with sections of varying ages, and the remaining 18% (8/45) were unsure. Most respondents (31/45) had Lamson (Kings Park, NSW, Australia) systems, while others had systems from Benchmark Systems (Willetton, WA, Australia), Aerocom (Thornleigh, NSW, Australia), Airlink Systems (Kilsyth, VIC, Australia), Telecom (Zwijndrecht, The Netherlands) and Swisslog (Redditch, United Kingdom). Hospitals were the primary managers of these systems, overseeing 73% (33/45), with pathology organisations managing 22% (10/45). Regarding transported items, blood samples were the most common at 96% (43/45), followed by paperwork at 93% (42/45), urine samples at 91% (41/45), blood cultures at 89% (40/45), and swabs at 89% (40/45). Although these are the totals, it should be remembered that a container may often contain mixtures of these.

Half of the respondents (23/45) were unsure how often their pneumatic tube systems were serviced (Table 1), and 63% (29/45) didn’t know if the systems were validated before installation (Table 2). Some validation methods included time-to-delivery, reliability, sample damage, and transport of different blood components and products. Some respondents referred to guidelines for validating systems, such as the AABB guidelines for pneumatic tube systems (*6*).

**Table 1:** Number and percentage of audit responses on how often the PTS is serviced

**How often is the pneumatic tube system serviced?**	**Responses (N = 45)**	**% Response**
Weekly	0	0
Never	2	4
Biannually	2	4
Monthly	6	13
Yearly	6	13
Quarterly	6	13
Unsure	23	51
PTS – pneumatic tube system.		

**Table 2:** Number and percentage of audit responses on whether the system has been validated as part of its installation

**Was your organization’s pneumatic tube system been validated as part of installation?**	**Responses (N = 45)**	**% Response**
Yes	14	31
No	2	4
Unsure	29	64

Most sites reported incidents involving their PTS (37/45, 80%), mainly system failures rather than sample loss, delays, or damage (Table 3). There was no relation between system age and incidents, though some respondents said ageing systems failed more often. There was also no trend between incident frequency and servicing (Table 4).

**Table 3:** Audit responses on the frequency of incidents involving pneumatic tube systems

	**Responses (N = 45)**				
	**< 5**	**5 to 10**	**> 10**	**Unsure**	**Other**
How many times has the pneumatic tube system failed in the past 6 months?	19	9	8	5	3
How many times have specimens been lost, misdirected or delayed when transported by the pneumatic tube system in the past 6 months?	26	2	5	10	0

**Table 4:** Comparison of responses on the frequency of servicing of pneumatic tube systems *versus* the number of incidents reported

**Response**	**Service interval of PTS**	**Failure of PTS in the past 6 months**	**Number of lost, misdirected or delayed specimens in the PTS in the past 6 months**	**Frequency of specimen transport delay through PTS, causing them to age (in the past 6 months)**	**Specimens have been going to the wrong location through PTS in the past 6 months**	**Number of specimens damaged in the PTS in the past 6 months (*i.e.* hemolyzed, clotted)**	**Frequency of specimen leakage in the system in the past 6 months**
1	3 monthly	< 5	< 5			< 5	< 5
2	6 monthly	Unsure	Unsure	< 5	< 5	Unsure	< 5
3	6 monthly	< 5	< 5				
4	Approx monthly?	42	Unsure	Unsure	Unsure	Unsure	< 5
5	Lamson service monthly, hospital staff do daily maintenance	Never fails, do have issues that are dealt with by the service team	< 5				
6	Monthly	5 to 10		5 to 10	5 to 10	Unsure	< 5
7	Monthly	> 10	< 5	< 5	< 5	Unsure	5 to 10
8	Monthly	Just around 40 times where the shift engineer was called in to fix (April- October	> 10				
9	Monthly						
10	Never	< 5	< 5				
11	Never	< 5	< 5				
12	Quarterly	5 to 10	< 5				
13	Quarterly	5 to 10	5 to 10	< 5	< 5	< 5	< 5
14	Quarterly	> 10	> 10	< 5	> 10	< 5	> 10
15	Quarterly	< 5	< 5			Unsure	Unsure
16	Quarterly	< 5	< 5			Unsure	Unsure

## Discussion

The lack of awareness of servicing and validation records suggests that laboratories may not have sufficient oversight of their operating systems. Instead, others often handle these responsibilities, such as hospital administration. Because of this, laboratories may not realise whether their systems are functioning correctly or can easily investigate issues such as turnaround times, which are crucial for improving their services. While the harm of delayed paperwork is not as severe as that of delayed blood samples or CSF, it may still impact patient care.

Something to note is that many respondents who were unsure whether their systems were validated or serviced also could not identify related incidents, further indicating the need for greater surveillance of laboratories.

Pneumatic tube systems pose a potential patient risk due to preanalytical errors, which can be mitigated through maintenance and vendor servicing adjustments tailored to each site (*1*). Greater oversight of laboratories is needed, and relationships with system vendors must be strengthened to ensure quality service. The results of the study primarily indicate that many laboratory respondents were unaware of servicing frequency, validation status and incident records, and that governance of PTS may be fragmented. However, another major finding of the study is that validation of the PTS has not been conducted, and that maintenance of these systems is not being undertaken. This is noted as anecdotal, based on the descriptive survey findings as represented in the individual responses in the Supplementary data.

There were limitations to this survey. As this was a survey-based audit, the results were subject to self-reporting bias and differences in respondents’ access to information on PTS servicing, validation and incident records. Also, access to service records, particularly shared PTS records, was not always available to respondents. For some incident categories, the numbers were small, reducing the statistical power of the findings. Finally, we acknowledge potential selection and response bias, as only 45 of 89 invited quality managers responded.

## Data Availability

All data generated and analyzed in the presented study are included in this published article (and its supplementary files).
